# Trends in Heavy Metal Pollution in Agricultural Land Soils of Tropical Islands in China (2000–2024): A Case Study on Hainan Island

**DOI:** 10.3390/toxics12120934

**Published:** 2024-12-23

**Authors:** Erping Shang, Yong Ma, Wutao Yao, Shuyan Zhang

**Affiliations:** 1 Key Laboratory of Earth Observation of Hainan Province, Hainan Aerospace Information Research Institute, Sanya 572022, China; 2 Aerospace Information Research Institute, Chinese Academy of Sciences, Beijing 100094, China

**Keywords:** Hainan Island, agricultural soils, heavy metal, trend

## Abstract

Heavy metal contamination in agricultural soils has garnered increasing attention, yet research on the spatiotemporal trends of heavy metal pollution in tropical regions with multiple annual crop harvests remains limited. This study examines data from 41 studies published between 2000 and 2024, including 206 records from 4122 sampling points on Hainan Island in China, to investigate the spatial distribution and temporal trends of heavy metal pollution. The results reveal that the average concentrations of Cd, Pb, As, Cr, and Hg in surface soil samples from agricultural lands on Hainan Island are 0.12, 28.28, 4.36, 63.98, and 0.075 mg/kg, respectively, all below the risk screening thresholds set by the Soil Pollution Risk Control Standard for Agricultural Land (GB 15618-2018). Spatially, heavy metal concentrations exhibit considerable regional variation. Cd levels are lower in the central region but higher in the northern and southern parts of the island. Both the cumulative pollution index and potential ecological risk index are elevated at the northern and southern ends, indicating more severe pollution in these areas. Pb and As show similar spatial patterns, with higher concentrations in the west and lower concentrations in the east. Conversely, Cr has higher concentrations in the northeast and lower concentrations in the southwest. Hg levels are elevated at the northern and southern ends of the island, though the overall pollution and ecological risk in these areas remain relatively low. Temporally, the concentration of heavy metals in agricultural soils has increased overall over the past two decades, with peak values occurring between 2017 and 2023. From 2002 to 2013, the variation was modest, while the largest fluctuations occurred between 2014 and 2016. Among the metals, Cr exhibited the most significant increase, indicating the most severe pollution, followed by Cd and Hg. As and Pb showed relatively lower levels of contamination. Regarding exceedance rates, the exceedances were evaluated against the thresholds established in GB15618-2018 and GB15618-1995. Cd’s exceedance rate increased from approximately 1% between 2002 and 2014 to between 7.78% and 20.93% in the following years, peaking in 2017. The exceedance rate for As rose slightly from 0% to 0.83%, with sporadic exceedances starting in 2015. Although these were relatively minor, a severe pollution point for As was observed in 2019. Exceedance rates for Pb and Cr increased significantly, from 0.75% and 7.50% in 2019 to 1.94% and 9.44% in 2023, reflecting increases of 4.8 to 10 times. These findings underscore the need for strengthened monitoring and management of heavy metal pollution in agricultural soils on Hainan Island to safeguard land quality and ensure the sustainability of local agricultural practices.

## 1. Introduction

Heavy metal contamination of agricultural land has become a global concern [1,2]. Heavy metals can be absorbed by crops, posing significant health risks to humans through the food chain [3,4,5,6]. Over the past two decades, research on heavy metal contamination in agricultural soils in China has increased [7,8,9,10]. However, the spatiotemporal dynamics of heavy metals in tropical agricultural regions remain insufficiently explored. In tropical areas, where triple cropping is common, understanding trends in soil heavy metal levels is essential for predicting future changes and developing effective strategies for assessing and managing heavy metal pollution.

Hainan Island, a tropical region in China, is one of the country’s least polluted areas. However, intensive agricultural practices, including triple cropping, alongside urbanization and tourism, have led to increased complexities in soil heavy metal accumulation patterns. While many studies have examined the spatial distribution, risk assessment, and pollution sources of heavy metals in Hainan’s soils [11,12,13], these studies often lack a comprehensive view of spatiotemporal trends. This gap largely results from the challenges associated with conducting long-term, continuous sampling over large geographic areas. As a result, meta-analysis [2,10], using data from the published literature, has emerged as an effective approach for tracking the dynamic changes in soil heavy metal levels on Hainan Island.

This study, therefore, aims to investigate the spatial distribution and temporal trends of heavy metal pollution in agricultural soils on Hainan Island, utilizing soil heavy metal data collected from studies published between 2000 and 2024. The findings of this research are of significant practical and scientific importance for promoting sustainable tropical agriculture in Hainan and mitigating the risks of soil heavy metal contamination to human health.

## 2. Materials and Methods

### 2.1. Overview of the Study Area

Hainan Island, situated in the southernmost region of China (18°10′–20°10′ N, 108°37′–111°03′ E) (Figure S1), represents the country’s largest tropical area. This region is characterized by the ability to harvest crops twice or even thrice annually, making it an ideal location for the development of efficient tropical agriculture. Hainan is particularly noted for its unique agricultural practices (such as seed base, characteristic agricultural products, ecological agriculture, and so on) and the cultivation of off-season fruits and vegetables [14].

### 2.2. Data Sources

**Figure 1:**
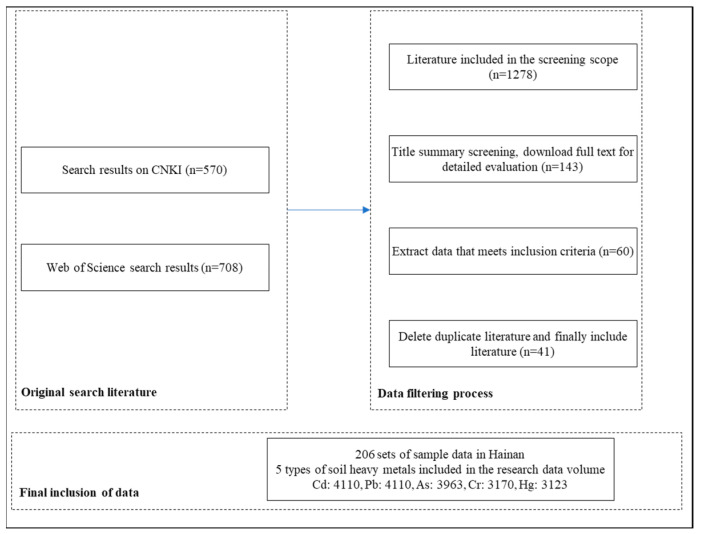
Data filtering process.

The primary source of heavy metal data is the literature, mainly obtained from databases such as CNKI and Web of Science. A hierarchical search was conducted to identify relevant papers on heavy metal pollution in agricultural soils on Hainan Island from 2000 to 2024. Search keywords included “Hainan”, “heavy metal”, “farmland”, “cultivated land”, “agricultural land”, “cadmium (Cd)”, “lead (Pb)”, “arsenic (As)”, “chromium (Cr)”, and “mercury (Hg)”. Initially, 1278 articles were reviewed, from which 143 were shortlisted based on titles and abstracts. After reviewing the full texts, 60 articles met the criteria, and duplicates were removed, leaving a final set of 41 papers (Figure 1, Table S1).

Data screening adhered to the following principles to ensure accuracy: (1) Samples must be from surface soils (0–15 cm or 0–20 cm); (2) The study area and location must be clearly described; (3) Sampling and analysis methods should comply with industry standards; (4) Heavy metal concentrations must include statistics such as mean, standard deviation, or the coefficient of variation; (5) Strict quality control protocols must be followed during sample preparation and analysis; and (6) Recognized analytical methods, such as atomic absorption spectroscopy, inductively coupled plasma mass spectrometry, must be used.

### 2.3. Data Extraction

The data extraction process primarily encompasses several key components: location information of the research area, details of sampling points, characteristics of farmland, soil pollutants, and surrounding environmental data.

- Research Area Information: Geographical coordinates (latitude and longitude), administrative ownership, and area size;
- Sampling Point Information: Sampling date (estimated as “publication year—2” if not recorded), number of samples, and location coordinates (inferred if necessary);
- Soil Pollutant Information: Heavy metal content, reported as mean values, ranges, standard deviations, coefficients of variation, or variances;
- Surrounding Environmental Information: Proximity to potential pollution sources, such as industrial or mining areas.

After thorough screening, total heavy metal (Cd, Pb, As, Cr, and Hg) data from 206 sets of 4122 agricultural soils in Hainan Island were extracted from 41 papers published between 2000 and 2024.

### 2.4. Calculation of Weighted Average

In field surveys, the size of the study area and the number of sampling points are crucial factors. Larger study areas with more sampling points and smaller variances tend to produce more reliable results and are thus given greater weight. The formula for calculating the weight $W_{i}$ is as follows:

(1) $$W_{i}=A_{i}\times N_{i}/Sd_{i},$$

where $A_{i}$ represents the size of the study area, $N_{i}$ denotes the number of soil samples, and $Sd_{i}$ signifies the calculated standard deviation of heavy metals in each study report.

The formula for calculating the weighted C-means is as follows:

(2) $$C=C_{i}\times W_{i}/\sum_{i=1}^{n}\;W_{i},$$

During the data inspection process, if the distribution of weights exhibits skewness, this can lead to an over-reliance on a few studies with extremely high weights. Log transformation is needed to address this issue. After log transformation, the distribution of weights becomes more similar to a normal distribution. The log-transformed weights ($W_{i}^{*}$) can be obtained through Equation (3), and the weighted average content (*C*) should be recalculated

(3) $$W_{i}^{*}=\mathrm{lg}\left(A_{i}\times \frac{N_{i}}{Sd_{i}}\right),$$

(4) $$C=C_{i}\times W_{i}^{*}/\sum_{i=1}^{n}\;W_{i}^{*},$$

The transformed weight coefficients exhibit a clear normal distribution in different regions, making the calculation results more representative.

### 2.5. Pollution Risk Evaluation Techniques

#### 2.5.1. Geological Accumulation Index Method

The geo-accumulation index ($I_{geo}$), developed in the 1960s [15], accounts for environmental geochemical background values and the impact of natural activities on these values. It is used to distinguish the influence of natural background levels versus anthropogenic pollution, providing a basis for identifying pollution sources. The calculation formula is as follows:

(5) $$I_{geo}=\log_{2}\left(\frac{C_{i}}{1.5B_{i}}\right)$$

$C_{i}$ represents the origin concentration of heavy metals in the soil samples, while $B_{i}$ denotes the natural background value of these metals. The coefficient of 1.5 is applied to account for potential variations in background values due to differences in geological formations across regions. A higher value of $I_{geo}$ indicates a greater severity of pollution. Specifically, when the I-geo value exceeds 0, it suggests that the heavy metals present in the soil are primarily derived from human activities rather than natural geological processes. The classification levels are summarized in Table 1:

#### 2.5.2. Potential Ecological Risk Index Method

The potential ecological risk index method links the environmental ecological effects of heavy metals with toxicology to assess their potential ecological risks [16].The calculation formula is as follows:

(6) $$E_{i}=T_{i}f_{i}=T_{i}\frac{C_{i}}{B_{i}}$$

where $E_{i}$ represents the single-element ecological risk index, $T_{i}$ is the toxicity coefficient with values, the toxicity coefficients for heavy metals Cr, Ni, Cu, Zn, As, Pb, and Cd are 2, 5, 5, 1, 10, 5, and 30, respectively [11], and $f_{i}$ is the ratio of the origin concentration $C_{i}$ of the heavy metal to its background value $B_{i}$. The classification of risk levels for $E_{i}$ and RI is detailed in Table 1.

## 3. Results and Discussion

### 3.1. Publication Bias

**Figure 2:**
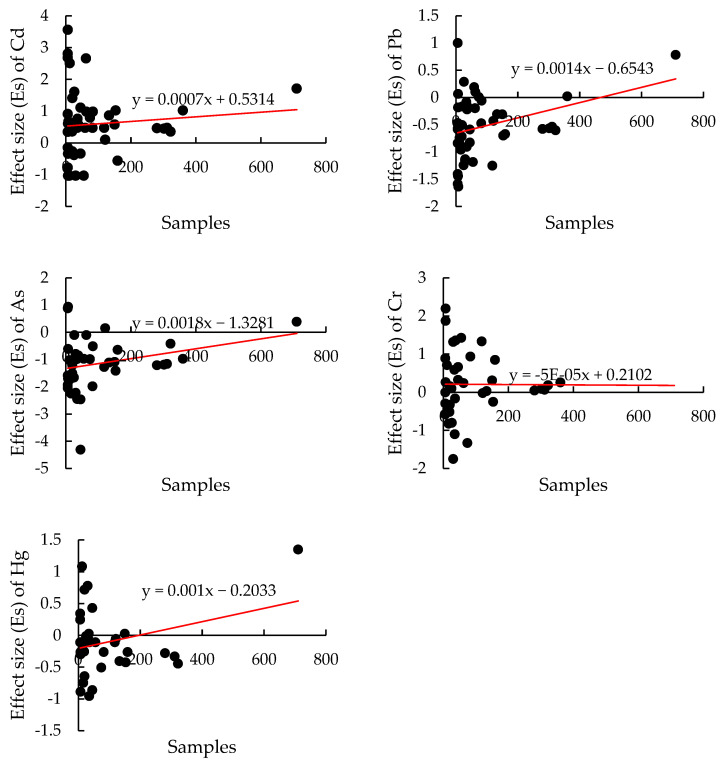
Publication bias.

To ensure the comprehensiveness of the literature review and the accuracy of the subsequent meta-analysis, a publication bias test was conducted. In this analysis, the effect size (Es) of heavy metals was treated as the dependent variable, with the sample size (n) as the independent variable. A linear regression model was used to assess the relationship between these variables. A slope close to zero in the regression line indicates minimal publication bias and higher data credibility. Figure 2 shows that the regression slopes for heavy metals range from 0.00005 to 0.001, which are near zero, suggesting low publication bias and confirming the reliability of the data for further analysis.

### 3.2. Heavy Metal Concentration in Soil of Agricultural Land in Hainan Island

The average sample-number-weighted concentrations of heavy metals (Cd, Pb, As, Cr, and Hg) in the surface soil samples were 0.12, 28.28, 4.36, 63.98, and 0.075 mg/kg, respectively (Table 2). Currently, it is common for the heavy metal content in farmland soils in Hainan to exceed local soil background values, indicating a certain degree of enrichment in the soil. Notably, the weighted average concentrations of Cd and Cr surpass the background levels in Hainan Province [17,18], with Cd concentrations being 2.18 times higher than the background value. Despite these elevated levels, the Cd sample-number-weighted content remains below the threshold of 0.3 mg/kg established by (GB15618-2018) [19]. This suggests that, although heavy metals have been introduced into agricultural soils on Hainan Island, their levels are generally within a safe range and pose minimal risk to agricultural product quality, crop growth, and the overall soil ecosystem. Furthermore, the average sample-number-weighted concentration of Cd is slightly lower than the 0.15 mg/kg level reported in agricultural soils on Hainan Island over the past five years [11,14]. This discrepancy can be attributed to the lower heavy metal content found in earlier soil samples. Additionally, the observed concentration is significantly lower than the Hg concentration (0.160 mg/kg) found in farmland soils across China, as reported by Song et al. [20].

### 3.3. Spatial Distribution Characteristics of Heavy Metal Content in Farmland Soil of Hainan Island

**Figure 3:**
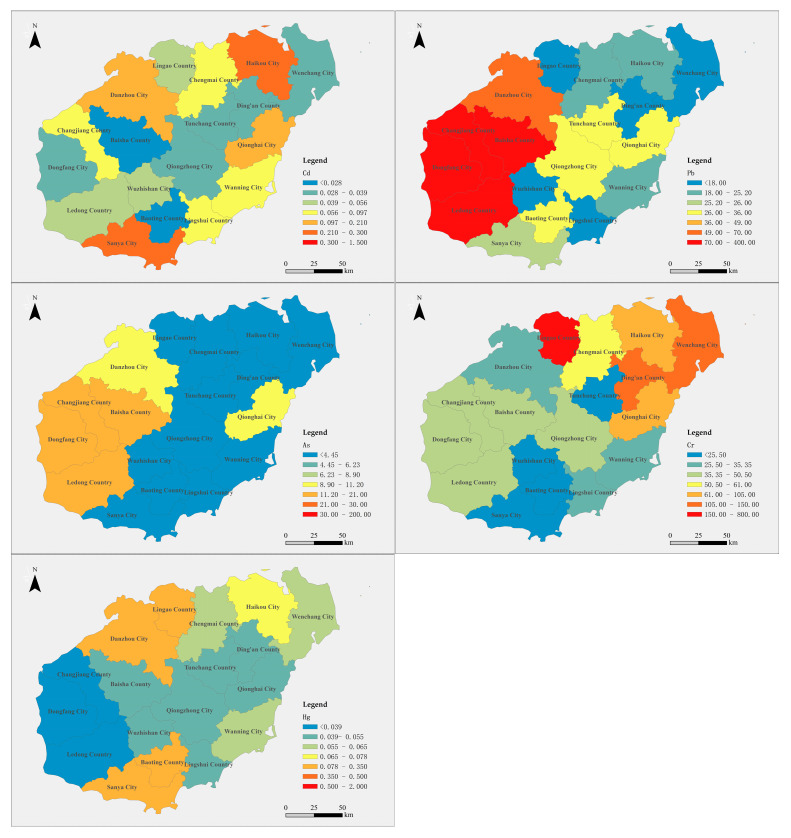
Spatial distribution of the sample-number-weighted concentrations in agricultural land soil of different cities in Hainan Island.

The spatial distribution of heavy metal sample-number-weighted concentrations in the agricultural soils across 18 cities and counties on Hainan Island is shown in Figure 3. In all of these areas, the sample-number-weighted concentrations of Cd, Pb, As, Cr, and Hg exceed the background values for either Hainan Province or national soil standards, with notable differences in the spatial distribution of these metals. Specifically, Cd concentrations display a trend of being lower in the central region and higher in the northern and southern parts of the island. The high-value areas are primarily located in the northern cities of Haikou and Danzhou, as well as the southeastern cities of Sanya and Qionghai, where the Cd concentrations are 2 to 4 times the background values.

Pb sample-number-weighted concentrations follow a west-to-east decreasing trend. In the western cities of Changjiang, Dongfang, Ledong, and Baisha, Pb levels exceed the risk screening values. In Danzhou, Pb concentrations are close to 70% of the risk screening value. In contrast, the eastern cities generally exhibit much lower Pb levels, with concentrations falling below 40% of the background value.

The distribution of As is similar to Pb, with higher concentrations primarily found in western cities, such as Dongfang, Ledong, Changjiang, and Baisha. However, the overall Pb concentrations in these areas are relatively low, with the As levels in most regions falling within the background value range for Hainan Province and all cities and counties remaining within the risk screening value.

The distribution pattern of Cr, however, contrasts with that of Pb and As, showing higher concentrations in the northeast and lower concentrations in the southwest. In the southwestern cities, Cr levels are generally below the background value of 50.5 mg/kg for Hainan Province, especially in Sanya, Baoting, and Wuzhishan, where Cr sample-number-weighted concentrations are less than half of the background value. The high-value area for Cr is in Lingao County, where the concentration exceeds the risk screening value but still remains within the control value range. Furthermore, the Cr concentrations in Wenchang and Dingan counties exceed the provincial background value, though they are still below the risk screening threshold.

Regarding Hg, none of the cities have Hg sample-number-weighted concentrations exceeding the risk screening value. Most cities show Hg levels below the background value of 0.078 mg/kg for Hainan Province, with relatively higher Hg concentrations (about 1 to 1.8 times the background value) observed in Sanya, Baoting, Lingao, and Danzhou. However, even in these areas, Hg levels remain within the safe range.

### 3.4. Assessment of Heavy Metal Pollution Risk in Agricultural Soils of Hainan Island

**Figure 4:**
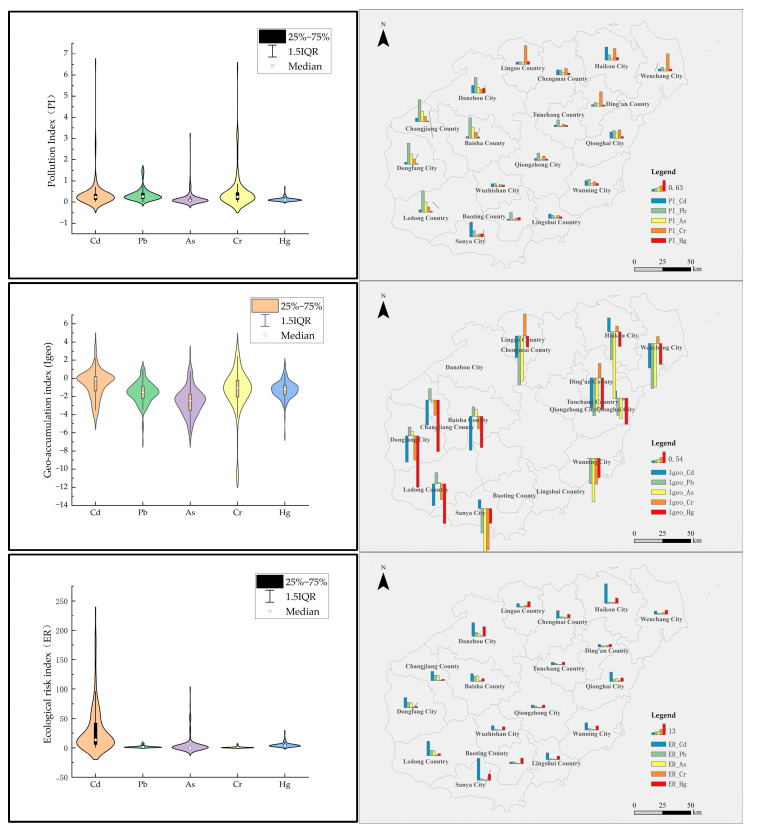
Hainan Island agricultural soil geo-accumulation index (Igeo) box plot and ecological risk index (ER) box plot.

An assessment of heavy metal contamination in the agricultural soils of the study area was conducted using the single-factor pollution index, the geo-accumulation index ($I_{geo}$), and the potential ecological risk index (ER). The results of this evaluation are shown in Figure 4.

From the perspective of the single-factor pollution index, the agricultural soils on Hainan Island are generally classified as clean, with some areas showing notable pollution from Cd and Cr. Overall, the levels of the five heavy metals in the central and southwestern parts of the island tend to have relatively low pollution indices. In contrast, the western regions exhibit relatively severe Pb pollution, particularly in Dongfang, Changjiang, Baisha, and Ledong, where the Pb pollution index exceeds 1. The northern areas show higher Cr pollution indices, while Cd pollution is relatively more pronounced at both the northern and southern ends of the island, especially in Lingao County in the north, where the pollution index exceeds 1. Hg concentrations are slightly higher in the southern city of Sanya and the northern city of Danzhou, but the single-factor pollution indices for Hg remain below 0.30, indicating relatively low overall pollution.

The geo-accumulation index (Igeo) for heavy metals reveals that most of the island is classified as either unpolluted or showing low to moderate pollution. Pb and As fall entirely into these two categories, with around 5% of the points showing moderate pollution, primarily in the western cities of the island. Cd, Cr, and Hg exhibit relatively heavier geo-accumulation pollution, with a certain proportion of moderate to high pollution levels ranging from 2.94% to 19.15%. Among these, Cd shows the highest levels of pollution, with 8.51% of points classified as high pollution and 2.13% as high-to-severe pollution, mainly located in the northern cities of Haikou and Danzhou, as well as in Sanya in the south. Cr has about 5.26% of points categorized as moderate-to-high pollution, concentrated in the northeastern cities, especially in Lingao County. Hg, with its highest pollution level classified as moderate, accounts for approximately 2.94% of the total, with these points mainly located in the northernmost and southernmost cities. Overall, the highest geo-accumulation pollution is found in the northern and southern extremes of the island for Cd, followed by Cr in the northeast and Hg at the island’s ends, while Pb and As show relatively light accumulation of pollution.

Regarding the potential ecological risk index, Pb, Cr, and Hg on Hainan Island are classified as posing slight ecological risk (Level I), indicating low risk. Although the risk values for Pb in the west, Hg in the north, and Cr in certain northern areas are relatively higher, they all remain below 40. As, with only 2.13% of its sampling points falling into moderate ecological risk (Level II), is mostly concentrated in the west. Cd poses a relatively higher risk, with 6.38% of points classified as strong ecological risk and 2.13% as very strong ecological risk (Level IV), particularly in Haikou and Danzhou in the north, and Sanya in the south. Overall, the potential ecological risks posed by the five heavy metals on Hainan Island are relatively low, with the exception of a few points with strong ecological risks (Level III) for Cd in the northern and southern extremes. For all other metals, the proportion of slight ecological risk (Level I) exceeds 97%.

In conclusion, while the overall heavy metal pollution on Hainan Island is relatively light, significant regional differences remain. The single-factor pollution index indicates that Pb pollution in the west, Cr pollution in the north, and Cd pollution at the island’s extremes are particularly notable and warrant attention. The geo-accumulation index further highlights Cd as the most serious pollutant, with major concentrations in Haikou, Danzhou, and Sanya. Additionally, the potential ecological risk index points to higher ecological risks from Cd, which could pose a threat to the local ecosystem. Despite the relatively low ecological risks from other metals such as Pb, Cr, Hg, and As, it is still essential to strengthen monitoring and preventive measures to prevent the further spread of pollution. These differences may be attributed to variations in soil physical and chemical properties, regional agricultural practices, industrial inputs, and environmental conditions. Therefore, targeted actions are required in Hainan Island’s heavy metal pollution control efforts to ensure the safety and sustainability of its ecological environment.

### 3.5. Trend of Soil Heavy Metal Concentrations

**Figure 5:**
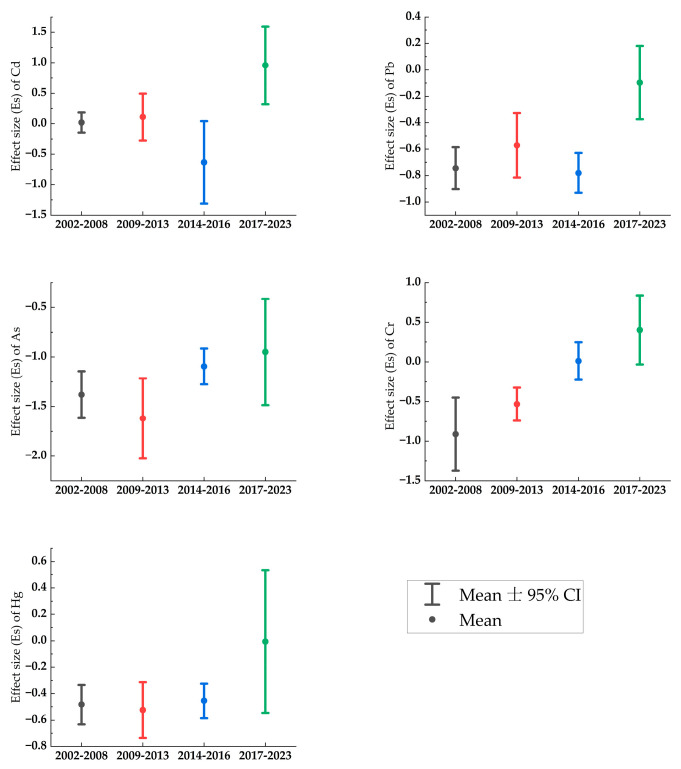
Trend of soil heavy metal concentrations in different time periods.

To characterize the temporal variations in heavy metal pollution in agricultural soils on Hainan Island, the collected data were grouped into four time periods: 2002–2008, 2009–2013, 2014–2016, and 2017–2023. This grouping allowed for an analysis of the changes in heavy metal pollution over time. Figure 5 illustrates the trends in heavy metal pollution in agricultural soils across Hainan Island. The results indicate that the heavy metal concentrations in Hainan Island’s agricultural soils show a fluctuating upward trend, with peak accumulation of Cd, Pb, As, Cr, and Hg occurring during the 2017–2023 period. This may be attributed to the cumulative effects of agricultural inputs (e.g., fertilizers and pesticides) and increased industrial emissions. Notably, the rise in heavy metal concentrations in recent years is more pronounced compared to earlier periods. However, the changes in different metals over time show slight variations as follows:

The effect value for soil Cr has shown a steady increase, with the mean effect value rising from −0.91 during 2002–2008 to −0.005 in 2017–2023. The peak effect value is 182 times greater than the trough value, indicating a significant upward trend and suggesting that Cr pollution has become more serious in recent years. The Cr peak may be linked to increased soil parent material, industrial discharges, the use of chromium-based agricultural inputs (e.g., pesticides), and its high mobility and low retention in acidic soils.

The changes in the effect values of Cd and Pb are quite similar. From 2002 to 2013, their effect values remained relatively stable. However, between 2014 and 2016, there was a marked decline, followed by a sharp increase in the 2017–2023 period. This suggests a significant rise in the input of Cd and Pb over the past five years, with Cd in particular showing a maximum effect value greater than 1.5, reflecting a considerable contribution from anthropogenic sources.

Arsenic concentrations showed a slight decline between 2009 and 2013 but experienced a significant rise during both the 2014–2016 and 2017–2023 periods. This may be attributed to variations in local soil pH, agricultural practices (e.g., phosphate fertilizer application), mining activities, industrial production, transportation, or other reasons. Although its effect value remains below zero, the upward trend in As pollution warrants attention, signaling a potential growing threat.

The changes in Hg concentrations were relatively stable from 2002 to 2016, but a sharp increase was observed between 2017 and 2023, signaling a rapid intensification of pollution during this period.

In summary, from 2002 to 2023, the concentrations of the five heavy metals have shown an overall increasing trend, with peak levels concentrated in the 2017–2023 period. The period from 2002 to 2013 showed relatively smooth changes, while 2014–2016 saw the greatest fluctuations. Among the five metals, Cr showed the most significant increase, with the highest effect values, indicating the most severe pollution. Following Cr, Cd and Hg also showed notable increases, while As and Pb exhibited relatively lighter pollution.

### 3.6. Trend of Heavy Metal Pollution Exceeding the Standard

In addition to comparing the temporal trends in the heavy metal effect values across different periods, this study also conducted a more thorough selection of the relevant literature based on strict criteria. The selection conditions were set as follows: the sampling range must cover the entire Hainan Island, and the sampling methods must be either uniformly or randomly distributed. After careful review, we identified several relevant studies that met these criteria, with sampling years primarily concentrated in seven years: 2005, 2009, 2014, 2017, 2019, and 2023. The number of samples collected each year ranged from 83 to 366.

**Figure 6:**
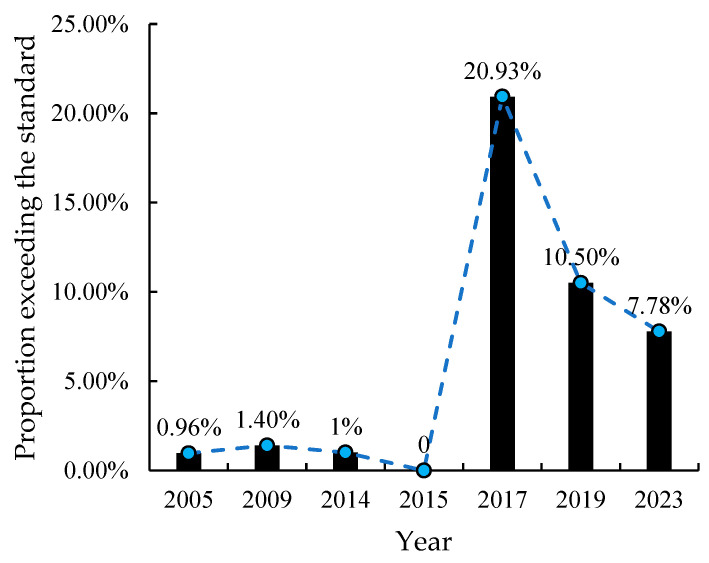
The proportion of excessive Cd pollution in agricultural soil on Hainan Island from 2005 to 2023.

**Figure 7:**
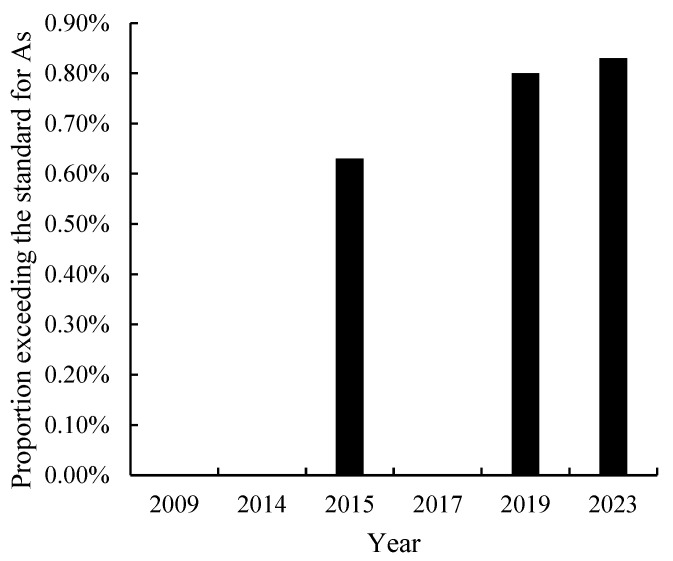
Trends in the proportion of excessive As pollution in agricultural soil on Hainan Island from 2009 to 2023.

**Figure 8:**
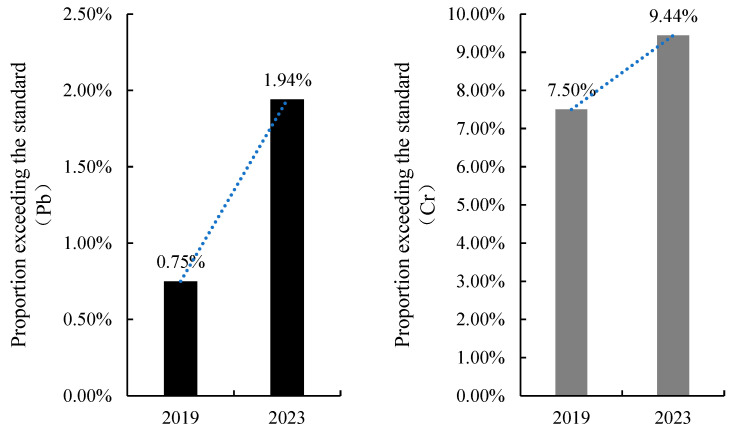
The proportion of excessive Pb and Cr pollution in agricultural soil on Hainan Island from 2019 to 2023.

During the in-depth analysis, we observed discrepancies in the threshold values for heavy metals between the GB15618-2018 [19] and the earlier Soil Environmental Quality Standards (GB15618-1995) [21]. To accurately assess the trends in heavy metal exceedance rates over time, we specifically selected metals for which the threshold values were the same in both standards for further study. By comparing the concentration changes of these heavy metals across different years and referencing the thresholds from the selected standards, we conducted detailed statistical analysis. The results of this analysis are presented in Figure 6, Figure 7 and Figure 8.

#### 3.6.1. Trend of Cd Pollution Exceeding the Standard

A stricter threshold of 0.3 mg/kg was commonly applied as the exceedance benchmark for Cd using the single-factor index method.

Over the past two decades, the exceedance rate of Cd has generally increased, with rates between 2017 and 2023 surpassing those from 2005 to 2014 by over sevenfold, highlighting a worsening pollution trend (Figure 6). Within these periods, distinct patterns emerge. Between 2005 and 2014, the exceedance rate remained relatively stable at around 1%, peaking at 1.40% in 2009 and dipping to 0.96% in 2005. In contrast, during 2017–2023, the rate declined from 20.93% in 2017 to 10.50% in 2019 and further to 7.78% in 2023. While this indicates some improvement in soil Cd levels, they remain significantly higher than two decades ago.

This pattern reflects two key factors. First, the rising exceedance rates between the two periods suggest that intensified agricultural activities and land use on Hainan Island have increasingly degraded soil quality, contributing to higher Cd contamination from 2017 to 2023 compared to 2005 to 2014. Second, the declining rates within the latter period point to the effectiveness of policy measures, such as the adoption of updated standards, in raising awareness and curbing further pollution. These findings emphasize the urgent need for sustained monitoring and targeted management strategies to mitigate heavy metal contamination and protect Hainan’s agricultural land.

#### 3.6.2. Trend of As Pollution Exceeding the Standard

During the period 2009 to 2023, different literature used 70 mg/kg as the threshold for evaluating soil As exceedance, and single-factor pollution assessments were conducted. It was found that, in the past two decades, the rate of arsenic exceeding the standard in soil has increased slightly from 0 to 0.83%. Before 2014, there were no soil As pollution sites, all of which were clean sites. However, after 2015, there were sporadic sites that exceeded the standard, but the proportion of exceeding the standard was relatively small. However, the highest soil As concentration (116 mg/kg) was in 2019, which was 3.86 times the threshold, and the site was classified as severely polluted. The second-highest value was observed in 2023, with a significant decrease in the max. value, which was 1.96 times the threshold and classified as mild but close to moderate pollution. Although the soil as a whole is mainly clean, the presence of heavily polluted sites also indicates the need to be vigilant about As pollution in the future.

#### 3.6.3. Trend of Pb and Cr Pollution Exceeding the Standard from 2019 to 2023

Pb and Cr had the same threshold in both 2019 and 2023, and their exceedance rates are shown in the Figure 8. The exceedance rate of Pb has increased rapidly, with the exceedance rate in 2023 being 2.6 times that of 2019, while the exceedance rate of Cr has only increased by 1.3 times. However, the exceedance rate of Cr each year is much higher than that of Pb, about 4.8–10 times higher.

## 4. Conclusions

The concentration of heavy metals in the surface soils of agricultural land on Hainan Island remains generally within safe levels. According to GB 15618-2018 [19], the average concentrations of Cd, Pb, As, Cr, and Hg are 0.12, 28.28, 4.36, 63.98, and 0.075 mg/kg, respectively, all of which fall below the regulatory threshold. However, spatial distribution patterns of these heavy metals exhibit distinct regional variations. The concentration of Cd is lower in the central part of the island but higher in the northern and southern regions. In contrast, Pb and As concentrations are notably higher in the western part of the island, with the highest levels of As concentrated in western urban areas. Chromium, on the other hand, has higher concentrations in the northeast and lower levels in the southwest. Mercury is generally found to be below the local background levels in most cities, except in Sanya, Baoting County in the south, and Lingao and Danzhou cities in the north, where it is slightly elevated, ranging from 1 to 1.8 times the background level but still within safe limits. The regional differences in heavy metal concentrations are caused by various factors, including atmospheric deposition, the mobility and availability of metals in soil, agricultural activities (such as fertilizer and pesticide use), industrial pollution, etc. In the future, we will focus on investigating the reasons for these geographic differences in metal concentrations and identifying sources of pollution to better understand and explain these differences, providing support for heavy metal pollution prevention and control.

Regarding pollution indices, the overall heavy metal contamination on Hainan Island is relatively low, though significant regional differences exist. The single-factor pollution index highlights that Pb pollution is more prominent in the west, Cr pollution in the north, and Cd pollution at both the northern and southern extremes. These areas warrant closer monitoring. The cumulative pollution index further underscores that Cd contamination is the most serious, with the highest levels found in Haikou and Danzhou in the north and Sanya in the south. Additionally, the potential ecological risk index indicates that Cd poses the highest ecological risk, potentially threatening local ecosystems. While the ecological risks associated with Pb, Cr, Hg, and As are relatively lower, ongoing monitoring and preventive measures are still necessary to prevent the further spread of contamination.

Over the past two decades, the concentrations of heavy metals in Hainan’s agricultural soils have generally shown an upward trend, particularly between 2017 and 2023, when peak values were observed. In contrast, from 2002 to 2013, the changes were relatively stable, and the most significant fluctuations occurred between 2014 and 2016. Among the heavy metals, Cr exhibited the most noticeable increase, with the highest pollution levels. Cd and Hg also showed significant rises, while As and Pb experienced relatively minor increases. In terms of exceedance rates, the rate for Cd rose from approximately 1% between 2002 and 2014 to 7.78%–20.93% between 2017 and 2023, peaking in 2017. The exceedance rate for As increased slightly from zero to 0.83%, with a few isolated exceedance points observed after 2015, though the exceedance was relatively minor. However, in 2019, severe As contamination was reported at specific sites. The exceedance rates for Pb and Cr also increased, from 0.75% and 7.50% in 2019 to 1.94% and 9.44% in 2023, reflecting a 4.8- to 10fold increase. These findings underscore the need for strengthened monitoring and management of heavy metal pollution in agricultural soils on Hainan Island to safeguard land quality and ensure the sustainability of local agricultural practices.

## Figures and Tables

**Table 1:** The evaluation criteria for *I*geo and $E_{i}$.

$I_{geo}$	Pollution Level	$E_{i}$	Ecological Risk
$I_{geo}$ ≤ 0	None	$E_{i}$ < 40	Low (I)
$0\;\text{<}\;I_{geo}$ ≤ 1	None to moderate	$40\;\text{≤}\;E_{i}$ < 80	Moderate (Ⅱ)
$1\;\text{<}\;I_{geo}$ ≤ 2	Moderate	$80\;\text{≤}\;E_{i}$ < 160	Considerable (Ⅲ)
$2\;\text{<}I_{geo}$ ≤ 3	Moderate to heavy	$160\;\text{≤}\;E_{i}$ < 320	High (Ⅳ)
$3\;\text{<}I_{geo}$ ≤ 4	Heavy	$E_{i}$ ≥ 320	Extremely high (Ⅴ)
$4\;\text{<}I_{geo}$ ≤ 5	Heavy to extreme	NA	NA
$I_{geo}$ > 5	Extreme	NA	NA

Abbreviations: NA represents not available.

**Table 2:** Comparison of the sample-number-weighted metal concentration in agricultural soil of Hainan (mg/kg).

Heavy Metals	Cd	Pb	As	Cr	Hg
Samples	4110	4110	3963	3170	3123
Min.	-	-	-	-	-
Max.	3.11	531.10	424.40	709.00	1.630
Mean (sample-number-weighted concentration)	0.12	25.43	3.87	61.38	0.067
Hainan Province background value [17]	0.056	36	8.9	50.5	0.078
China background value	0.097	26.00	11.20	61.00	0.065
Risk screening value (GB15618-2018) [19]	0.3	70	30	150	0.5
Risk control value (GB15618-2018) [19]	1.5	400	200	800	2

## Data Availability

The original contributions presented in the study are included in the article. Further inquiries can be directed to the corresponding author.

## Supplementary Materials

The following supporting information can be downloaded at: https://www.mdpi.com/article/10.3390/toxics12120934/s1, Figure S1: Location map of the research area; Table S1: The references for meta-analysis.
