# Supplementary material for: Trends in Heavy Metal Pollution in Agricultural Land Soils of Tropical Islands in China (2000–2024): A Case Study on Hainan Island
Source: Toxics. 2024 Dec 23;12(12):934. doi: 10.3390/toxics12120934 (PMC11728724; doi:10.3390/toxics12120934)
Supplement: Supplementary file 1 [file toxics-12-00934-s001.zip › toxics-3371166-supplementary.pdf]

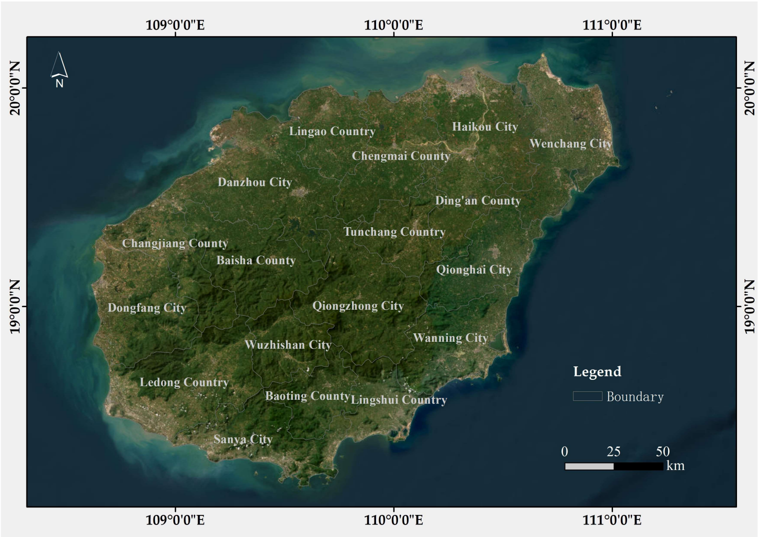

Figure S1. Location map of the research area.

Table S1. The references for meta-analysis.

ID	References
1	Chen, W.; Wang, P.; Xiong, C. Evaluation and Zoning of Cultivated Land Soil Quality Based on the Comprehensive Quality Impact Index. Shanghai Land & Resources 2021, 42(3), 77–84.
2	Xu, S.; Wang, H.; Fu, S. Distribution Characteristics and Source Apportionment of Heavy Metals in Farmland Soil of Hainan Island Based on Receptor Models and Geostatistics. Environmental Pollution & Control 2021, 43(9), 1164–1169.
3	Zhang, J.; Li, C.; Xie, Y.; Meng, L. Evaluation of Irrigation Water and Soil Quality in Orchards in Hainan. Journal of Tropical Agriculture Science 2020, 40(9), 13–19.
4	Liang, J.; Sun, H.; Ge, C.; Meng, L. Distribution and Health Risk Assessment of Heavy Metal Content in Soils from Major Agricultural Production Areas of Hainan Province. Chinese Journal of Tropical Crops 2019, 40(11), 2285–2293.
5	Li, J.; Li, X.; Ge, C.; Yu, H.; Sun, H.; Chen, M. Human Health Risk Assessment in Areas with High Natural Background Levels of Heavy Metals in Soil in Qiongbei, Hainan. Chinese Journal of Tropical Crops 2018, 39(1), 189–196.
6	Li, X.; Li, J.; Sun, H.; Yu, H.; Ge, C. Levels of Heavy Metals and Potential Ecological Risks in Farmland Soils in Qiongbei, Hainan. Journal of Agro-Environment Science 2017, 36(11), 2248–2256.
7	Liu, Y.; Lin, Y.; Huang, S.; Tang, Q.; Zhang, M.; Fu, J.; Wu, L. Distribution Characteristics and Evaluation of Heavy Metal Pollution in Soils of Hainan Agricultural Reclamation Areas. Journal of Tropical Agriculture Science 2017, 37(7), 10–16.
8	Liu, Y.; Lin, Y.; Huang, S., et al. Characteristics and Assessment of Heavy Metal Content in Soils from Tropical Crop Fields in Hainan Island. Guangdong Agricultural Sciences 2017, 44(7), 59–64.
9	Zhou, B.; Qin, X.; Yuan, Q. Study on the Characteristics of Heavy Metal Content in Vegetable Soils in Haikou. Environmental Protection Science 2016, 42(5), 124–128.
10	Tong, X.; Mo, F.; Liu, F.; Tang, X.; Guo, B. Heavy Metal Content and Assessment of Rural Soils in Haikou. Journal of Hainan Normal University (Natural Science) 2015, 28(2), 186–189.
11	He, Y.; Liao, X.; Ni, Q.; Wang, F.; Fu, X. Distribution Characteristics and Pollution Assessment of Heavy Metals in Soils and Vegetables of Vegetable Bases in Haikou. Soil Bulletin 2015, 46(3), 721–727.
12	Wang, C.; Luo, S. Distribution and Assessment of Heavy Metal Content in Suburban Vegetable Bases in Haikou. Journal of Tropical Agriculture Science 2016, 36(8), 62–68.
13	Guo, B.; Tong, X.; Wei, Z. Preliminary Study on Heavy Metal Pollution in Paddy Soils of Lingshui County. China Tropical Agriculture 2015, 1, 73–77.

14 Xiao, Y.; Luo, S.; Wang, Y.; Yu, Y.; Huang, W.; He, Y. Selenium, Cadmium, and Lead Accumulation in Vegetables from Chengmai and the Effects of Selenium. Environmental Science & Technology 2014, 37(8), 76–79.
15 Li, F.; Li, X.; Yang, F.; Qi, Z. Evaluation and Source Analysis of Heavy Metal Pollution in Farmland Soils of Hainan Island. Journal of Hainan University (Natural Science) 2013, 31(3), 211–217.
16 Li, F. Investigation and Evaluation of Heavy Metal Pollution in Agricultural Land Soils of Hainan Island. Master's Thesis, Hainan University, 2010.
17 Li, F.; Li, X.; Wu, P.; et al. Correlation between Heavy Metal Content, Soil Organic Matter, and pH in Agricultural Land Soils of Hainan Province. Soil 2009, 41(1), 49–53.
18 Han, C.; Song, X.; Li, J.; Ren, H.; Zhao, C.; Wu, D.; Wang, L.; Bai, Z. Spatial Distribution Characteristics of Trace Elements in Cultivated Soils of Sanya. Journal of Hainan Normal University (Natural Science Edition) 2013, 26(3), 274–278.
19 Geng, J.; Wang, W.; Wen, C.; Yi, Z.; Tang, S. Selenium and Heavy Metal Content, Distribution, and Safety in Paddy Soils of Hainan. Acta Ecologica Sinica 2012, 32(11), 3477–3486.
20 Guo, Y.; Fu, Y.; Bai, X.; Yang, Y.; Zhang, G. Evaluation and Source Analysis of Heavy Metals in Soils of Pepper Plantations in Hainan. Soil Bulletin 2012, 43(3), 711–717.
21 Zhao, Z.; Wang, P.; Zhao, G.; Wang, J. Study on the Enrichment of Heavy Metals in Surface Soils of Agricultural Lands in Western Hainan Island. Ecological Environment Journal 2012, 21(1), 136–139.
22 Li, F.; Li, X.; Wu, P.; et al. Spatial Distribution and Pollution Assessment of Heavy Metals in Fruit and Vegetable Soils in Sanya. Ecological Journal 2010, 29(2), 382–386. DOI:10.13292/j.1000-4890.2010.0020.
23 Lv, L.; Guo, B.; Qi, Z. Preliminary Study on Heavy Metal Pollution in Paddy Soils of Wanning City, Hainan. Journal of Tropical Crops 2009, 30(7), 1023–1027.
24 Zhao, Z.; A. W. Rate; Tang, S.; Bi, H. Spatial Distribution and Environmental Significance of Heavy Metals in Agricultural Land Soils of Hainan Island. Journal of Agricultural Environment Science 2008, 1, 182–187.
25 Zhu, W.; Yang, Y.; Bi, H.; Liu, Q. Study on the Content and Bioavailability of Zn, Pb, Cu, and Cd in Hainan Soils. Mineralogical Journal 2004, 3, 239–244.
26 Li, J. Spatial Distribution and Health Risk Assessment of Heavy Metals in Soils of High Background Areas in Northern Qiongbei. Master's Thesis, Hainan University, 2018.
27 Jiang, W. Ecological Geochemical Assessment of Potential Harmful Elements in Agricultural Soils in the Beibu Gulf Region. PhD Dissertation, China University of Geosciences (Beijing), 2015.
28 Zhong, P. Identification and Distribution of Human Inputs of Heavy Metals in Hainan Agricultural Land Soils. Master's Thesis, Hainan University, 2015.
29 Han, M. Heavy Metal Distribution in Agricultural–Rice Systems in Western Hainan. Master's Thesis, Hainan University, 2014.
30 Wu, J.; Wu, C.; Wang, J. Ecological Risk Assessment and Source Apportionment of Heavy Metals in Agricultural Soils in Haikou. Journal of Tropical Crops 2023, 44(5), 1062–1071.
31 Zhang, Y.; Kuang, J.; Xie, Y.; et al. Soil Environmental Quality Evaluation of Agricultural Land in Hainan Province. Journal of Subtropical Resources and Environmental Sciences 2014, 9(3), 75–81.
32 Gong, Q.; Yang, J.; Wang, Z.; et al. Migration Characteristics of Heavy Metals in Soil–Tea Systems in Qiongzhong County, Hainan, and Health Risk of Tea Consumption. Geophysical and Geochemical Exploration 2023, 47(3), 826–834.
33 Xie, Y. Soil Environmental Quality Evaluation in Fruit Production Areas of Hainan Province. Journal of Tropical Agriculture Science 2017, 37(11), 39–47.
34 Wei, Z.; Guo, B.; Qi, Z. Heavy Metal Pollution in Rice Soils of Wenchang City, Hainan Province. Anhui Agricultural Science 2009, 37(35), 17616–17619.
35 Zuo, W.; Li, Q.; Zhang, Y.; et al. Heavy Metal Pollution and Health Risk Assessment in Agricultural Land in the Volcanic Weathering Zone: A Case Study of Jiangdong New District, Haikou. Earth Science 2024, 1–15.
36 Huang, H.; Su, H.; Li, X.; Li, Y.; Jiang, Y.; Liu, K.; Xie, X.; Jia, Z.; Zhang, H.; Wang, G.; Ye, Z.; Cheng, X.; Wen, J.; Li, N.; Yu, Y. A Monte Carlo Simulation-Based Health Risk Assessment of Heavy Metals in Soils of the Tropical Region in Southern China. Environmental Geochemistry and Health 2024, 46(7), 234. DOI:10.1007/s10653-024-02021-3.

37 Lin, W.; Zhang, Y. Investigation and Evaluation of Heavy Metal Pollution in Suburban Vegetable Gardens of Wuzhishan City, Hainan. Agricultural Environment and Development 2007, (3), 101–102.
38 Yu, Y. GIS-Based Spatial Distribution and Pollution Evaluation of Heavy Metals in Soils of Hainan Island. Master's Thesis, Hainan Normal University, 2014.
39 Hao, L. GIS-Based Evaluation of Heavy Metal Pollution in Agricultural Land Soils of Hainan Island. China Science and Technology Investment 2013, (11), 41–44.
40 Wu, Y.; Xia, Y.; Mu, L.; Liu, W.; Wang, Q.; Su, T.; Yang, Q.; Milinga, A.; Zhang, Y. Health Risk Assessment of Heavy Metals in Agricultural Soils Based on Multi-Receptor Modeling Combined with Monte Carlo Simulation. Toxics 2024, 12, 643. DOI:10.3390/toxics12090643.
41 Jiang, W.; Hou, Q.; Yang, Z.; Yu, T.; Zhong, C.; Yang, Y.; Fu, Y. Annual Input Fluxes of Heavy Metals in Agricultural Soil of Hainan Island, China. Environmental Science and Pollution Research International 2014, 21(13), 7876–7885. DOI:10.1007/s11356-014-2679-0.
